# Systemic high-dose MTX and BTK inhibitors in vitreoretinal lymphoma: efficacy and survival analysis

**DOI:** 10.1097/BS9.0000000000000279

**Published:** 2026-02-03

**Authors:** Jing Gao, Li-wei Lv, Suo-wang Zhou, Na Yao, Lei Yang, Jin Ye, Lu Zhang, Xiaoyan Peng, Liang Wang

**Affiliations:** a Department of Hematology, Beijing Tongren Hospital, Capital Medical University, Beijing, China; b Department of Ophthalmology, Beijing Tongren Hospital, Capital Medical University, Beijing 100730, China; c Institute of Hematology, Union Hospital, Tongji Medical College, Huazhong University of Science and Technology, Wuhan, China

## 1. INTRODUCTION

Vitreoretinal lymphoma (VRL) is a rare subtype of central nervous system lymphoma (CNSL) that predominantly comprises B-cell lymphomas. Because of its clinical resemblance to uveitis and transient response to glucocorticoid therapy, the diagnosis of VRL is often delayed.^1^ Although initial manifestations appear indolent, the disease can lead to irreversible vision loss or central nervous system (CNS) relapse, resulting in a poor prognosis.^2^ Based on the site of initial malignant lymphocyte infiltration, primary VRL (PVRL) originates in the vitreous or retina without CNS or systemic involvement, whereas secondary VRL (SVRL) arises from CNSL or systemic lymphoma.^3^

As the most common form of intraocular lymphoma, VRL poses substantial therapeutic challenges because of its anatomical constraints and the high risk of CNS progression, a key determinant of long-term survival outcomes.^4^ The therapeutic management of VRL remains controversial because no universally accepted standard of care has been established. Local therapies, including intravitreal methotrexate (IVT-MTX) injections and radiotherapy, are commonly used to control intraocular disease; however, these modalities fail to prevent CNS dissemination, the primary driver of disease-related mortality.^4^

Systemic therapies, particularly high-dose methotrexate (HD-MTX)–based regimens, have been investigated to reduce CNS progression by eradicating occult systemic or CNS-infiltrating tumor cells.^5^ Retrospective studies suggest that HD-MTX decreases the rate of CNS progression in VRL, although its efficacy and toxicity profiles require further validation in VRL-specific cohorts.^5^ Bruton’s tyrosine kinase (BTK), a non-receptor kinase critical for B-cell receptor signaling, plays a central role in the pathogenesis of B-cell lymphomas.^6^ BTK inhibitors (BTKis), such as ibrutinib, have demonstrated efficacy in CNSL. Soussain et al and our institution reported that BTKi monotherapy achieves high response rates in VRL, potentially owing to the pharmacological and physiological similarities between the blood-brain and blood-retinal barriers.^7–9^ Nevertheless, clinical evidence for BTKi use specifically in VRL remains limited, and large-scale studies evaluating the combination of systemic HD-MTX and BTKi are lacking.

Because VRL is rare, most reports are limited by small sample sizes and single-center designs. We conducted a retrospective analysis of 56 patients with VRL treated at 2 centers in China to provide more robust evidence. We aimed to assess the impact of therapeutic approaches, including IVT-MTX, HD-MTX–based systemic therapy, and BTKis, on outcomes such as progression-free survival (PFS), overall survival (OS), and CNS progression. We intend for these findings to inform clinical decision-making by clarifying the role of systemic prophylaxis in the multidisciplinary management of this malignancy.

## 2. METHODS

We analyzed 56 patients diagnosed with VRL between August 2019 and July 2025, including 51 patients from Beijing Tongren Hospital, Capital Medical University, and 5 patients from Union Hospital, Tongji Medical College, Huazhong University of Science and Technology. The cohort included 47 patients with PVRL, 2 with primary CNSL (PCNSL) who later developed ocular involvement, and 7 with concurrent PCNSL and VRL at diagnosis. Treatment approaches were heterogeneous and often overlapping. Eighteen patients received IVT-MTX alone. Twenty-nine received HD-MTX–based systemic therapy, potentially combined with a CD20 monoclonal antibody or a BTKi. Thirty patients received a BTKi. Of the 30 patients treated with a BTKi, 22 received it in combination with HD-MTX for CNS prophylaxis, 7 received a BTKi without HD-MTX, and 1 received a BTKi with other chemotherapeutic agents. Primary endpoints were treatment response, PFS, OS, and the incidence of CNS progression. Secondary endpoints included ocular relapse, brain relapse, ocular-free survival (OFS), and brain-free survival (BFS).

At diagnosis, all patients underwent comprehensive systemic evaluation, including brain magnetic resonance imaging (MRI), whole-body positron emission tomography/computed tomography (PET-CT), diagnostic vitrectomy, and measurement of interleukin-10 (IL-10) and interleukin-6 (IL-6) levels in the vitreous or aqueous humor. Ophthalmic examinations were performed using a slit-lamp biomicroscope. Lumbar puncture was performed for cerebrospinal fluid (CSF) analysis, including cell count, protein and glucose concentrations, cytology, and flow cytometry. Based on these findings, CNS involvement was confirmed, determining the final diagnostic classification. Treatment regimens were individualized. In patients receiving systemic therapy, HD-MTX was administered with leucovorin rescue, often in combination with rituximab. IVT-MTX was typically administered twice weekly for 4 weeks, followed by weekly injections for 4 additional weeks, and then monthly for 3 months.

Ophthalmic evaluations were performed at 2 weeks and at 1, 2, and 5 months, alongside serial brain MRI monitoring. Treatment response was assessed based on ophthalmic and neuroimaging findings. Complete response (CR) was defined as the complete disappearance of intraocular lymphoma on ophthalmic examination and the absence of CNS disease on MRI. OS was calculated from the date of diagnosis to the date of death or last follow-up. PFS was calculated as the time from diagnosis to disease relapse or progression (irrespective of site) or death from any cause. Ocular relapse was defined as the reappearance of disease or emergence of new lesions in the anterior chamber, vitreous, or retina after achieving a CR or partial response (PR) to first-line therapy. Brain relapse was defined as the detection of CNSL confirmed by CT, Fluorodeoxyglucose PET/CT, or MRI, or by pathological assessment, following an initial response. CNS progression was defined as any new CNS involvement occurring during the course of VRL treatment. BFS and OFS were measured as the time from diagnosis to brain and ocular relapse, respectively. Patients who were alive and event-free were censored at the last follow-up visit.

Survival curves were generated using the Kaplan–Meier method, and the cumulative incidence of CNS progression was estimated. Risk factors for OS were analyzed using Cox proportional hazards regression. Age, sex, bilaterality, systemic therapy, BTKi treatment, corticosteroid use, ocular relapse, and CNS/systemic relapse were evaluated. Variables were categorized using predefined reference levels. Univariate analyses were conducted for all variables, and those with a *p* value <0.200 were included in the multivariate model. Binary logistic regression was used to identify clinical factors associated with relapse, including ocular and CNS/systemic relapses. Variables considered in the logistic regression models were consistent with those in the Cox regression analysis and were categorized similarly. Results are presented as odds ratios (ORs) and hazard ratios (HRs) with 95% confidence intervals (CIs). The Chi-square test was used to compare the incidence of CNS progression between patients who received HD-MTX and those who did not. Among HD-MTX–treated patients, the Fisher exact test was used to assess the association between BTKi treatment and relapse. Forest plots were generated using the ggplot2 package in R (version 4.1.1; R Foundation for Statistical Computing, Vienna, Austria), with HR estimates represented by points and 95% CIs by horizontal bars on a logarithmic scale. The study protocol was approved by the institutional review boards of Beijing Tongren Hospital (TREC2022-KY103).

## 3. RESULTS

### 3.1. Study characteristics and treatments

This study included 56 patients with VRL (25 men and 31 women). The median age at diagnosis was 56 years (range, 35–79). Twenty-nine patients (51.8%) presented with bilateral ocular involvement, and 18 (32.1%) had previously received corticosteroid therapy. Histopathological classification identified 54 cases of B-cell lymphoma, 1 case of marginal zone lymphoma, and 1 case of T-cell lymphoma. One patient presented with B symptoms. Clonal Immunoglobulin kappa light chain and Immunoglobulin heavy chain gene rearrangements were detected in the vitreous fluid of 19 patients. A definitive diagnosis via vitrectomy and cytopathology/histopathology was confirmed in 25 patients, whereas 31 were diagnosed based on a combination of clinical and laboratory findings, such as an elevated IL-10/IL-6 ratio in the ocular fluid.^10^ An IL-10/IL-6 ratio >1.0 was observed in the vitreous fluid, aqueous humor, and CSF of 7, 27, and 27 patients, respectively. Monoclonal B cells were detected by CSF flow cytometry in 4 cases. One patient with normal renal function showed elevated serum ß2-microglobulin levels. Patient characteristics and first-line treatment protocols are summarized in Table 1.

**Table 1:** Patients characteristics and initial treatments.

Patient demographics	Number of patients (%)
Age, median (range), y	56 (35–79)
Sex	
Male	25 (44.6)
Female	31 (55.4)
Bilaterality	
Yes	29 (51.8)
No	27 (48.2)
Diagnosis level	
Cytologic diagnosis	25 (44.6)
Cytokine (IL-10/IL-6 ratio) + clinical evidence	31 (55.4)
Median time to diagnosis (range), d	11 (0–524)
Disease involvement	
Eye	47 (83.9)
CNS	2 (3.6)
Eye and CNS	7 (12.5)
Median best corrected visual acuity of affected eyes at diagnosis (13 eyes received intraocular MTX)	0.3 (0.01–1)
Vitreous haze at diagnosis (17 patients)	13 (76.5)
Lumbar puncture performed	45 (80.4)
IL-10 and IL-6 cytokines in the aqueous humor (39 eyes)	
IL-10 level (pg/mL): median (range)	197.9 (1–26,803.4)
Elevated IL-10 (>30 pg/mL)	30 (76.9)
IL-10/IL-6 ratio (>1)	33 (84.6)
Initial treatment	
IVT-MTX only	17 (30.4)
HD-MTX–based chemotherapy only	13 (23.2)
BTKi only	2 (3.6)
IVT-MTX and BTKi	5 (8.9)
IVT-MTX and HD-MTX–based chemotherapy	16 (28.6)
IVT-MTX and other chemotherapy	1 (1.8)
Other therapy	2 (3.6)
Intravitreal injection of MTX treatment course	8 (0–41)
HD-MTX treatment course	4 (3–6)
Total treatment course	6 (1–17)
Follow-up period, median (range), d	593 (5–2183)

BTKi = Bruton’s tyrosine kinase inhibitor, CNS = central nervous system, HD-MTX =high-dose intravenous methotrexate, IL = interleukin, IVT-MTX = intravitreal methotrexate.

### 3.2. Tumor response and survival

Five patients were not evaluable for treatment response: 3 because of missing data, 1 who sought treatment elsewhere, and 1 who could not be assessed because of COVID-19. Among the 51 evaluable patients, 35 (68.6%) achieved CR, 15 (29.4%) attained PR, and 1 (2.0%) had progressive disease (PD) with systemic progression. Following IVT-MTX monotherapy, vision improved in 8 of 13 treated eyes (61.5%), with a median visual acuity of 0.6 (range, 0.05–1.0). One eye experienced visual deterioration caused by cataract formation. After completion of first-line therapy, among 41 evaluable patients, 26 (63.4%) achieved CR, 11 (26.8%) attained PR, and 3 (7.3%) had PD.

**Figure 1.**
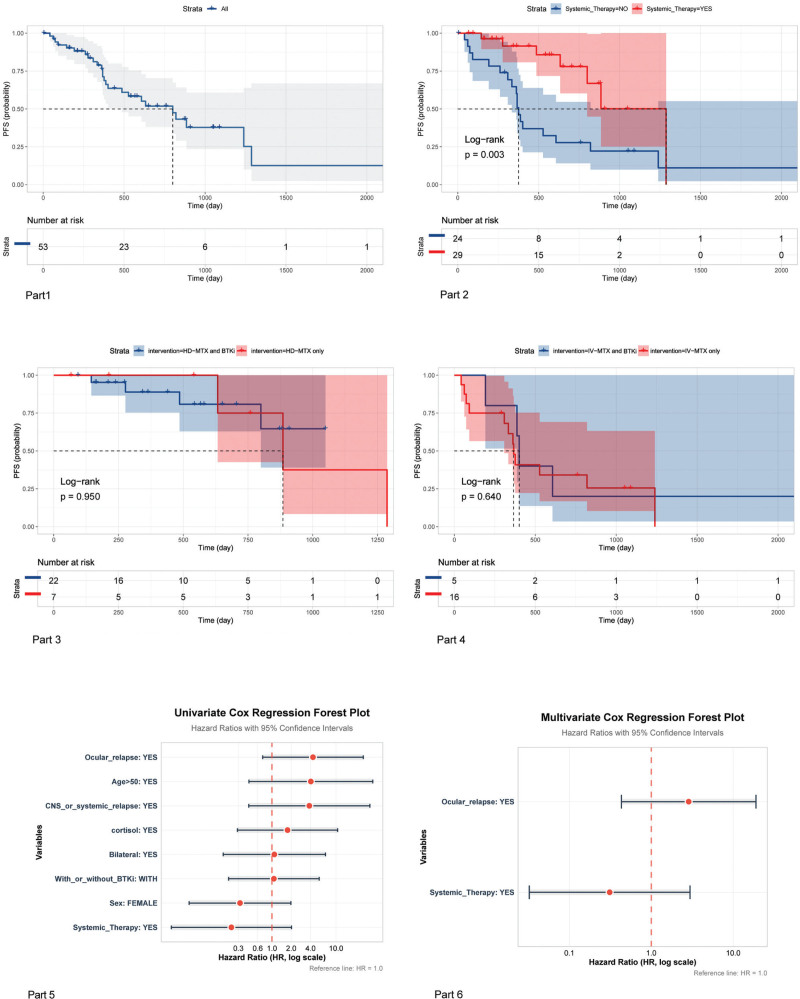
Survival outcomes and treatment effects in patients with primary vitreoretinal lymphoma. Part 1: Kaplan–Meier curve of PFS for the entire cohort; Part 2: Comparison of PFS between patients receiving HD-MTX–based systemic therapy and those who did not; Part 3: Comparison of PFS with vs without BTKi therapy in patients receiving HD-MTX–based regimens; Part 4: Comparison of PFS with vs without BTKi therapy in patients receiving IVT-MTX–based regimens; Part 5: Forest plot of univariate analysis for OS; Part 6: Forest plot of multivariate analysis for OS. BTKi = Bruton’s tyrosine kinase inhibitor, CNS = central nervous system, HD-MTX = high-dose methotrexate, IVT-MTX = intravitreal methotrexate, mPFS = median progression-free survival, OS = overall survival, PFS = progression-free survival.

Survival data were available for 53 patients, with a median follow-up of 593 days (3 were lost to follow-up). The median PFS (mPFS) was 800 days (95% CI: 486–NR) (**Fig. 1**, Part 1). The 1-, 2-, and 5-year PFS rates were 76.3%, 51.8%, and 12.6%, respectively. Patients who received HD-MTX–based systemic therapy demonstrated significantly longer PFS than those who did not (*p* = 0.003; **Fig. 1**, Part 2). The mPFS was 1287 days (95% CI, 800 days–not reached) in the HD-MTX group and 376 days (95% CI, 334–820 days) in the non–HD-MTX group. Two patients treated with BTKi monotherapy achieved CR and PR, with PFS durations of 367 and 261 days, respectively; both later developed CNS progression. Across the full cohort, BTKi therapy did not significantly improve PFS (*p* = 0.170), and no benefit was observed when BTKis were combined with either HD-MTX (*p* = 0.950; **Fig. 1**, Part 3) or IVT-MTX (*p* = 0.640; **Fig. 1**, Part 4). Similarly, among patients receiving HD-MTX, no significant difference in PFS was observed between those who received IVT-MTX and those who did not (*p* = 0.290). Other clinical factors, including age, sex, bilaterality, and prior corticosteroid use, had no significant effect on PFS. Patients with SVRL demonstrated a trend toward longer PFS than those with PVRL (*p* = 0.087), although this did not translate into a statistically significant difference in OS (*p* = 0.340). This PFS trend may be explained by the more intensive combination regimens used in SVRL cases. Subgroup analysis of patients with PVRL revealed that HD-MTX use was associated with significantly longer mPFS than non-use (1287 vs 376 days; *p* = 0.019). In contrast, BTK inhibition did not confer a significant PFS benefit in this subgroup (*p* = 0.470). The median OS was not reached, with a projected 5-year OS rate of 47.9% (95% CI: 0.191–1). In Cox regression analysis, univariate models indicated that older age and disease relapse were nominally associated with worse OS (HR >1), whereas systemic therapy and female sex were associated with improved OS (HR <1). After multivariate adjustment, ocular relapse retained a nominally increased hazard and systemic therapy had a nominally reduced hazard; however, neither reached statistical significance (*p* > 0.050), likely reflecting limited statistical power. Summary results are illustrated in Figure 1, Parts 5–6.

### 3.3. Toxicity and relapse

Among 38 patients who developed treatment-related toxicities, 14 (36.8%) had grade I to II myelotoxicity, and 2 (5.3%) had grade III to IV events. Hematologic toxicities included neutropenia (31.6%), thrombocytopenia (18.4%), and anemia (34.2%). Non-hematologic events included transaminase elevation (21.1%) and creatinine elevation (15.8%). Of 39 patients receiving IVT-MTX, ocular toxicities included keratitis (n = 4), cataract (n = 5), elevated intraocular pressure (n = 1), and optic disc edema (n = 1). No ocular complications were related to anterior chamber paracentesis, and no systemic chemotherapy-related ocular toxicity occurred. No treatment-related deaths or therapy discontinuations occurred because of adverse events. During follow-up, 25 of 53 patients (47.2%) relapsed, involving the CNS (n = 20), ocular (n = 5), and systemic (n = 2) sites. Specifically, 18 patients had CNS-only relapse, 3 had ocular-only, 1 had systemic-only, 2 had concurrent ocular and CNS relapse, and 1 had ocular and CNS involvement. Among 47 patients with PVRL, 19 (40.4%) experienced CNS progression. The incidence of CNS progression was significantly lower in patients who received HD-MTX than in those who did not (17.2% vs 62.5%; *p* = 0.001). The median BFS was significantly longer in the HD-MTX group (1341 days; 95% CI, 885 days–not reached) than in the non–HD-MTX group (401 days; 95% CI, 367 days–not reached) (*p* = 0.006). Ocular relapse was less frequent than brain relapse, and the median OFS was not reached. Among 29 HD-MTX–treated patients, relapse occurred in 3 of 7 (42.9%) who did not receive a BTKi vs 2 of 22 (9.1%) who did (*p* = 0.074). None of the 3 patients receiving first-line HD-MTX plus a BTKi followed by hematopoietic stem cell transplantation relapsed; their longest PFS to date is 705 days.

### 3.4. Treatment of the relapses

In the second-line setting, 20 of 25 relapsed patients (80%) received HD-MTX–based chemotherapy. Among them, 18 received a CD20 monoclonal antibody (rituximab or zuberitamab), and 16 were additionally treated with a BTKi (orelabrutinib or zanubrutinib). Among evaluable second-line patients, 12 achieved CR, 3 achieved PR, 2 had stable disease (SD), and 3 had PD. Five were not evaluable or were pending assessment. Seven patients proceeded to third-line therapy (1 PR, 3 PD, others pending evaluation), and 1 patient received fourth-line Chimeric Antigen Receptor T-Cell Immunotherapy, achieving PR. Multivariate logistic regression identified systemic therapy as an independent protective factor against CNS or systemic relapse (OR = 0.109, 95% CI: 0.018–0.490; *p* = 0.007). For ocular relapse, systemic therapy also showed a protective trend (OR = 0.130; *p* = 0.119). In contrast, corticosteroid use was identified as a significant risk factor for CNS or systemic relapse (OR = 8.573, 95% CI: 1.465–75.455; *p* = 0.030). No other clinical variables—including age, sex, bilaterality, BTKi use, or primary site—were significantly associated with relapse outcomes.

## 4. DISCUSSION

Our retrospective analysis of 56 patients reaffirms the pivotal role of systemic HD-MTX–based therapy in improving PFS and reducing CNS progression, consistent with the broader literature and our previous meta-analyses. In the present cohort, HD-MTX was associated with significantly prolonged median PFS (*p* = 0.003) and a markedly lower CNS progression rate (17.2% vs 62.5%; *p* = 0.001). These results align with those of our earlier systematic review that highlighted the importance of systemic therapy in extending PFS and OS, particularly in preventing CNS relapse, the leading cause of mortality in patients with VRL.^11^

Although IVT-MTX remains the cornerstone of local disease control, our data suggest that it is insufficient as a standalone strategy to prevent CNS infiltration. This observation aligns with the findings that local therapy alone was associated with high rates of intraocular and CNS recurrences.^12^ It has also been demonstrated that although IVT-MTX, rituximab, and local radiotherapy could eradicate intraocular lymphoma cells, these localized treatments failed to prevent subsequent CNS relapse.^4^ In contrast, the integration of systemic HD-MTX appears to offer a prophylactic benefit, potentially by eradicating microscopic systemic or CNS disease that is undetectable at diagnosis. However, its ability to control intraocular disease appears to be less robust. In our cohort, a subset of patients experienced ocular relapse despite systemic therapy, mirroring the findings of the French LOC network study, where HD-MTX–based chemotherapy alone was associated with a high ocular relapse rate (58%).^5^ This suggests that although HD-MTX penetrates the blood-ocular barrier, intravitreal drug levels may be insufficient to eradicate residual vitreoretinal disease. Supporting this hypothesis, we observed that combined local and systemic therapy was associated with improved ocular control, underscoring the complementary role of systemic prophylaxis and local disease eradication.

Our cohort achieved a favorable median OS, with a projected 5-year OS rate of 47.9%. This may be attributed to 2 factors: first, the effective prevention of CNS progression through upfront HD-MTX, and second, the successful application of salvage therapies at relapse. In our study, all patients who experienced relapse received systemic salvage therapy. These second-line regimens included HD-MTX–based chemotherapy combined with other agents such as rituximab, BTKis, temozolomide, or lenalidomide. A subset of patients underwent hematopoietic cell transplantation. This treatment pathway underscores the importance of subsequent therapy intensification to achieve long-term survival. Regarding BTKi therapy, our data did not demonstrate a significant PFS benefit with monotherapy, consistent with our earlier meta-analysis that reported a lower CR rate for BTKis alone than for combination regimens.^13^ Future studies should explore whether BTKis enhance the efficacy of HD-MTX using a combinatorial approach, particularly for high-risk VRL.

Our study also identified HD-MTX–based systemic therapy as an independent protective factor against CNS/systemic relapse (OR = 0.109, *p* = 0.007), reinforcing the notion that CNS-directed systemic treatment is critical for VRL management. Furthermore, our data suggest a clinically relevant reduction in CNS relapse rates with the HD-MTX and BTKi combination compared with HD-MTX alone, a benefit not reflected in the PFS analysis. This raises the question of whether combination therapy offers a distinct protective advantage against CNS-specific progression. Conversely, prior corticosteroid use was associated with an increased risk of CNS relapse, which may reflect delayed diagnosis or masked disease activity, underscoring the need for early and definitive treatment.

The safety profile observed in our cohort was manageable, with no treatment-related deaths or discontinuations. Ocular adverse events related to IVT-MTX were consistent with those reported in previous studies, and systemic toxicities were predominantly low-grade. This favorable profile can be largely ascribed to stringent monitoring and proactive management during HD-MTX administration, including hydration, alkalinization, drug level monitoring, and leucovorin rescue. Consequently, our data substantiate the feasibility of integrating systemic HD-MTX into VRL therapeutic strategies.

Our study has several limitations. Its retrospective design, small sample size, and the heterogeneity of treatment regimens limit the ability to draw definitive conclusions. Additionally, the follow-up duration may have been insufficient to capture late relapses or long-term toxicities. Furthermore, the nonrandomized allocation of treatments introduced potential selection bias. Nevertheless, our findings are strengthened by detailed clinical data that provide real-world insights into VRL management.

In conclusion, this study reinforces the importance of systemic HD-MTX therapy for improving PFS and reducing CNS progression in patients with VRL. Although HD-MTX alone may not fully prevent ocular relapse, its combination with local therapy and novel agents, such as BTKis, represents a promising strategy to enhance disease control. These results support the integration of CNS-directed systemic therapy into the first-line management of VRL, particularly for patients with primary ocular disease who are at high risk of CNS relapse. Prospective randomized trials are needed to validate these findings and optimize combinatorial strategies for this malignancy.

## ACKNOWLEDGMENTS

Funding received from Noncommunicable Chronic Diseases-National Science and Technology Major Project (2025ZD0544300).

## AUTHOR CONTRIBUTIONS

J.G., L.-w.L., and S.-w.Z. collected the data. J.G. wrote the manuscript and performed data analysis. N.Y., L.Y., and J.Y. reviewed the manuscript. L.Z., X.P., and L.W. contributed to study conceptualization, data acquisition, and approved the final version.
